# Oromucosal Administration of a Cannabidiol‐Enriched *Cannabis* sp. Extract for 2 Weeks Moderately Reduces Cold Hyperalgesia in Rats With Neuropathic Pain

**DOI:** 10.1002/ejp.70307

**Published:** 2026-06-05

**Authors:** Raquel Maria Pereira Campos, Luciana Conde, Andrey Aguiar, Luzia Sampaio, Ricardo Augusto de Melo Reis, Pedro Moreno Pimentel‐Coelho

**Affiliations:** ^1^ Carlos Chagas Filho Biophysics Institute Federal University of Rio de Janeiro Rio de Janeiro Brazil; ^2^ D'or Institute for Research and Education (IDOR) Rio de Janeiro Brazil

**Keywords:** cannabidiol, cannabis, endocannabinoid system, immune cells, microglia, neuropathic chronic pain

## Abstract

**Background:**

Chronic neuropathic pain (CNP) involves complex interactions between resident and peripheral immune cells, as well as modulation of the endocannabinoid system. Cannabis‐based products have emerged as promising therapeutic options, but their effects on immune cells remain unclear. This study evaluated the effects of a cannabidiol (CBD)‐enriched *Cannabis* sp. extract administered via the oral mucosa in a CNP model.

**Methods:**

Two‐month‐old Wistar rats of both sexes were randomly assigned to four groups. Two groups underwent chronic constriction injury (CCI) of the sciatic nerve, while the remaining groups underwent sham surgery (SHAM). Starting at Day 7 post‐CCI, rats received either the CBD‐enriched extract (10 mg/kg) or vehicle for 15 days. Mechanical and thermal sensitivity were assessed using the von Frey, acetone and hot plate tests. Ipsilateral spinal cord segments were analysed by flow cytometry, immunohistochemistry and western blotting.

**Results:**

CCI induced thermal hyperalgesia and mechanical allodynia. Treatment with the CBD‐enriched extract modestly reduced cold sensitivity in CCI rats of both sexes but did not improve heat sensitivity and had a significant but minor effect on mechanical thresholds. CCI increased spinal cord microglial cell numbers, particularly in females, an effect not modulated by treatment. No significant changes were detected in immune cell infiltration or CB1R, CB2R, or TRPV1 protein expression across groups.

**Conclusion:**

Oromucosal treatment with a CBD‐enriched extract modestly alleviates cold hypersensitivity in both sexes. Neuroimmune alterations in the spinal cord at 22 days post‐CCI indicate a predominance of microglia, with no detectable peripheral immune cell infiltration, regardless of treatment.

**Significance Statement:**

This manuscript contributes to addressing gaps in the literature regarding preclinical models evaluating *Cannabis* sp. based products using treatment protocols that more closely reflect patient use, as commercially available cannabidiol‐enriched extract and the oromucosal route, consistent with the route commonly used by patients. In addition, all experiments were conducted in animals of both sexes, addressing a well‐recognised limitation of many preclinical studies in chronic pain research. Notably, the International Association for the Study of Pain has emphasised the need for further research in this area before endorsing the use of *Cannabis* sp. based products for the treatment of chronic pain.

## 1 Introduction

Chronic Neuropathic Pain (CNP) is defined by the International Association for the Study of Pain as persistent pain resulting from a lesion or disease affecting the somatosensory nervous system (Nicholas et al. 2019). It affects approximately 7%–10% of the global population and substantially reduces quality of life (van Hecke et al. 2014; Scholz et al. 2019). CNP arises from diverse causes, including metabolic disorders and mechanical injuries, and is characterised by symptoms such as mechanical hyperalgesia and thermal allodynia (Alles and Smith 2018).

The pathophysiology of CNP involves complex cellular and molecular mechanisms. Increasing evidence highlights the role of immune components, including peripheral immune cells, microglia and cytokines. After nerve injury, spinal microglia undergo phenotypic changes and release factors that promote neuronal hyperexcitability (IInoue and Tsuda 2018). T lymphocytes also infiltrate the spinal cord within 3–7 days following injury in rodent models, contributing to neuroinflammation (Cao and DeLeo 2008; Costigan et al. 2009; Li et al. 2020). Additionally, sex differences influence CNP mechanisms, with distinct immune and glial contributions observed in males and females (Midavaine et al. 2021).

CNP treatment remains challenging. First‐line therapies include gabapentinoids and opioids, which often show limited efficacy and carry risks such as dependence and overdose (Eisenberg and Suzan 2014; Hennemann‐Krause and Sredni 2016; Campos et al. 2021). Consequently, safer and more effective alternatives are needed. The medicinal use of Cannabis sp. dates back to ancient China (Bonini et al. 2018). The plant produces numerous metabolites, primarily flavonoids, terpenes and phytocannabinoids. Δ9‐tetrahydrocannabinol (THC) and cannabidiol (CBD) are the most studied. THC acts as a partial agonist at cannabinoid receptors and is responsible for psychotropic effects, whereas CBD interacts with multiple molecular targets, including FAAH, 5‐HT1A receptors and TRPV1 (Patricio et al. 2020). The combined actions of cannabinoids and other plant constituents may produce an ‘entourage effect’ (Russo 2011, 2019). Clinical and observational studies suggest that cannabis‐based products can reduce pain, improve quality of life and decrease opioid requirements (Nurmikko et al. 2007; Ueberall et al. 2019; Nielsen et al. 2022; Bicket et al. 2023). Common formulations include CBD:THC 1:1 ratios or THC‐rich products, often alongside other Cannabis components (Ueberall et al. 2019, 2022; Bar‐Lev Schleider et al. 2022).

In this study, we administered a commercially available CBD‐enriched Cannabis extract daily via the oromucosal route to rats with CNP induced by chronic constriction injury (CCI), mimicking the route of administration used in patients. We evaluated behavioural pain outcomes and assessed spinal immune cell populations and endocannabinoid system (EcS) modulation. This clinically relevant model provides translational insight into how Cannabis‐based therapies may influence neuroimmune mechanisms underlying CNP.

## 2 Materials and Methods

### 2.1 Animals

Two‐month‐old male and female Wistar rats were housed (maximum of 4 animals/cage) under a 12‐h light–dark cycle (dark cycle from 6 a.m. to 6 p.m.) at a controlled room temperature (23*°*C *±* 1*°*C), with free access to water and food, and without environmental enrichment. A total of 172 rats were used (88 males and 84 females). All procedures followed the National Institutes of Health Guidelines for the Care and Use of Laboratory Animals and were approved by the Ethics Committee for the Use of Animals in Research of the Federal University of Rio de Janeiro (CEUA CCS protocol #93/20).

### 2.2 Chronic Constriction Injury (CCI)

CNP was induced by CCI of the right sciatic nerve (Austin et al. 2012). Rats were anaesthetised with a combination of ketamine hydrochloride (500 mg/Kg) and xylazine hydrochloride (50 mg/Kg), administered intramuscularly into the hind limb. After 5 min, anaesthesia was assessed by pinching the hind paw with a tweezer. If the animals responded, an additional half‐dose of the anaesthetic was administered. Animals that did not respond were placed in the dorsal position, the fur was removed from the hindlimb, and the area disinfected with 70% ethanol. A skin incision was made in the thigh to reach the muscular fascia, followed by separation of the gluteal muscles and biceps femoris muscle to expose the sciatic nerve. Constriction was performed using 6–0 silk suture, with 3 ligatures tied around the sciatic nerve at 3 mm intervals. The sciatic nerve was repositioned, and muscles and skin were sutured with nylon thread. In the sham‐operated control group, the sciatic nerve was exposed but not constricted.

### 2.3 Experimental Design

**FIGURE 1:**
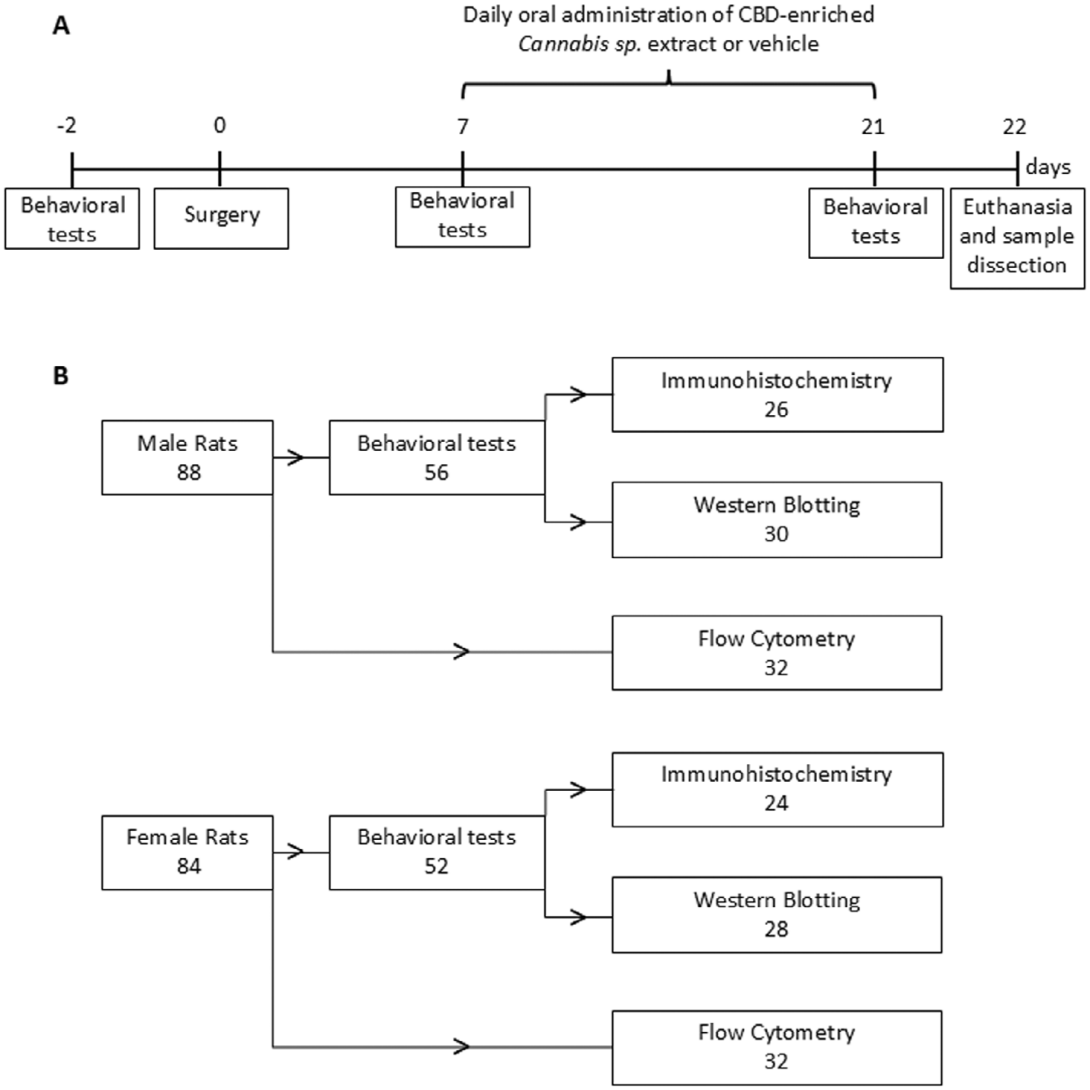
Experimental design and animal allocation. (A) Experimental design timeline and (B) flowchart of the number of animals used in each experiment organised by sex.

Rats of both sexes were randomly assigned to four groups: Sham + Vehicle, Sham + Extract, CCI + Vehicle and CCI + Extract. Behavioural assessments of mechanical and thermal sensitivity were conducted 2 days before surgery (baseline) and on Days 7 and 21 post‐surgery. Starting on Day 7 post‐surgery, rats received daily oral administration of either a CBD‐enriched *Cannabis* sp. extract or a medium‐chain triglyceride (MCT) oil solution (vehicle). On Day 22 following surgery, the animals were euthanised (Figure 1A). The first animals subjected to behavioural testing were subsequently used for spinal cord sample collection for Western blotting, whereas the remaining animals were used for immunohistochemical analyses. Rats used for flow cytometry analysis were not subjected to behavioural testing. A flowchart showing the number of animals used in each experiment, stratified by sex, is shown in Figure 1B.

### 2.4 Cannabis Extract Component Analysis and Cannabis Extract Administration

A commercially available CBD‐enriched Cannabis sp. extract and its corresponding resuspension vehicle (MCT oil) were obtained in accordance with Brazilian sanitary regulations. The phytocannabinoid profile of the extract was assessed and quantified for internal quality control, as shown in Table 1. Phytocannabinoids were extracted from the commercial product using methanol: n‐hexane (9:1 v/v), followed by ultrasonication for 10 min and centrifugation for a further 10 min at 2000×*g*. The resulting supernatant was filtered through a 0.22 μm membrane and analysed by high‐performance liquid chromatography with photodiode array detection (HPLC‐PDA). Chromatographic separation was performed on a C18 column (250 mm × 4.6 mm i.d., 5.0 μm) maintained at 30°C, using a gradient mobile phase consisting of 50 mM ammonium formate buffer (pH 5.19) and methanol at a flow rate of 1.0 mL/min, and quantification was carried out at 240 nm (Carvalho et al. 2020).

**TABLE 1:** Main phytocannabinoids concentrations in the CBD‐enriched *Cannabis* sp. extract evaluated by liquid chromatography.

	CBD	CBDA	THC	THCA	CBN
Mean concentration (mg/mL)	17.15	0.43	0.06	0.00	0.00
Percentage composition	97.22	2.44	0.34	0.00	0.00

All animals were housed at university facilities and were handled by experimenters for 2–3 days prior to the start of the experiments to reduce stress associated with manipulation and immobilisation. Animals were weighed every 4–5 days for dose adjustments. The extract was administered at a dose of 10 mg/kg/day of CBD, corresponding to a THC dose of 0.035 mg/kg/day, once daily during the dark phase of the circadian cycle. Vehicle groups received MCT oil. The oromucosal route was chosen to better reflect clinical practice and to reduce discomfort and stress associated with oral gavage (da Costa Rodrigues et al. 2025). For administration, animals were gently restrained by lifting the skin at the back of the neck, preventing tail contact with the cage and minimising aversive responses. The viscous Cannabis sp. extract or MCT oil was then delivered to the inner cheek using a micropipette (volumes ranged from 80 μL to 120 μL depending on animal weight and increased by approximately 20 μL over the 2‐week treatment period).

### 2.5 Behavioural Tests

All behavioural assessments were performed on the same day in the following order: Von Frey (mechanical sensitivity), acetone test (cold sensitivity) and hot plate test (heat sensitivity), with a 20‐min interval between tests. Animals were placed individually in acrylic enclosures (12 × 20 × 17 cm) with wire mesh flooring and acclimated for 30 min under red light prior to testing.

#### 2.5.1 Von Frey Test

After the acclimation period, mechanical sensitivity was assessed using Von Frey filaments (Stoelting Co.). An ascending stimulus protocol was used, starting with the 0.07 g filament. Five stimuli, each separated by at least 1 min, were applied to the plantar surface of the right hind paw. If the animal showed at least 3 positive responses (paw withdrawal), the force exerted by that filament was considered as the mechanical threshold (Deuis et al. 2017).

#### 2.5.2 Acetone Test

The acetone test was conducted to measure thermal sensitivity to cold. Rats remained in the von Frey apparatus, and the test began 20 min after the completion of the Von Frey test. A 100 μL jet of acetone was sprayed on the plantar surface of the right hind paw. Animals were observed for 2 min, and responses were scored as follows: 0 (no response), 1 (quick withdrawal or paw movement at the time of acetone application), 2 (repeated paw shaking) and 3 (repeated paw shaking and paw licking) (Deuis et al. 2017).

#### 2.5.3 Hot Plate Test

After the acetone test, animals were returned to their home cages, and the hot plate test was performed 20 min later to assess thermal sensitivity to heat. Each animal was placed individually on a hot plate set at 52°C ± 1°C, and the latency to right hind paw withdrawal, paw licking, or jumping was recorded. A cut‐off time of 30 s was applied to prevent injury from prolonged exposure of the paws to the thermal stimulus (Deuis et al. 2017).

### 2.6 Tissue Dissection

Animals were euthanised and transcardially perfused using a perfusion pump on Day 22 post‐surgery. For flow cytometry and western blotting, perfusion was performed with 0.9% saline, and the ipsilateral (right) L4‐L6 spinal cord segments were dissected. For immunohistochemistry, perfusion was continued with 4% paraformaldehyde, after which the L4‐L6 segments were collected for further processing.

### 2.7 Flow Cytometry

**FIGURE 2:**
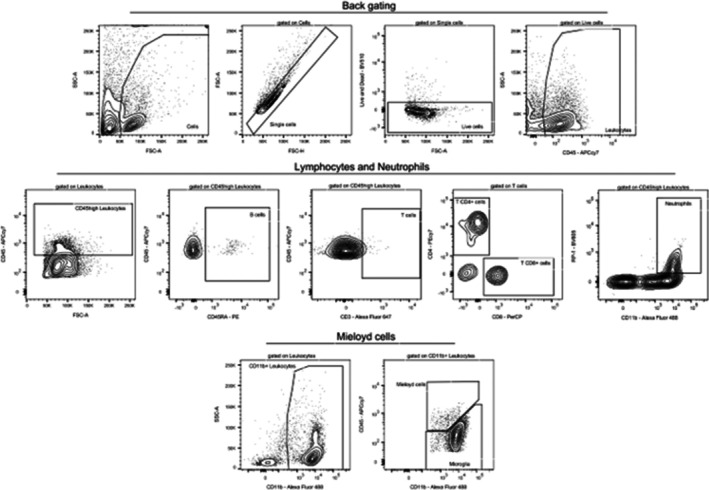
Flow cytometry gating strategy for identification of immune cell populations in the spinal cord. Representative flow cytometry plots illustrating the gating strategy used to identify myeloid cells (CD45^high^CD11b^+^ myeloid cells and CD45^low^CD11b^+^ microglia), neutrophils (CD45^high^CD11b^+^RP1^+^), CD4^+^ and CD8^+^ T cells (CD45^high^CD3^+^CD4^+^ and CD45^high^CD3^+^CD8^+^) and B cells (CD45^high^CD45R^+^) in the spinal cord.

Spinal cord tissue was minced and subjected to enzymatic digestion using 2 mg of collagenase D (Sigma Aldrich, #11088858001) per sample in FACS buffer (phosphate‐buffered saline + 5% fetal bovine serum) for 40 min at 38°C. The samples were then passed through a 70 μm cell strainer (Biofil, #CSS013070) and centrifuged at 1500 rpm for 5 min. The resulting pellet was resuspended in FACS buffer for washing and centrifuged again at 1500 rpm for 5 min. After removing the supernatant, the pellet was resuspended in a solution containing 37% Percoll (Cytivia, #17544501) and centrifuged at 400 g for 30 min with no acceleration or brake. Cells were washed as described above and then resuspended in a staining mix containing monoclonal antibodies conjugated to fluorochromes (Table 2) for 45 min at 4°C. Following a final wash, cells were resuspended in FACS buffer for acquisition on a BD LSRFortessa flow cytometer (BD Bioscience). Cell populations were gated and analysed using FlowJo software (BD; v7.10.01) (Figure 2).

**TABLE 2:** Antibodies and viability dye used in the flow cytometry experiments.

Live and dead (BV510)	#L34957—life technologies
anti‐CD11b (FITC)	#55331—BD Pharmigen
anti‐CD4 (PECy7)	#201515—Biolegend
anti‐CD8 (Percep)	#201712—Biolegend
anti‐CD3 (APC)	#557030—BD Bioscience
anti‐CD45 RA (PE)	#202307—Biolegend
anti‐CD45 (APC Cy7)	#561586—BD Bioscience
anti‐RP1 (BV605)	#743055—BD Bioscience

### 2.8 Western Blotting

Dissected ipsilateral (right) spinal cord segments were stored at −80°C until sample preparation. Tissues were sonicated in lysis buffer (50 mM HEPES, 1 mM MgCl2, 10 mM EDTA, 1% Triton X‐100, pH 6.4) containing a protease inhibitor cocktail (1:100, Sigma‐Aldrich, P2714‐1BTL). Supernatants were collected after centrifugation at 10.000 rpm for 10 min at 4°C, and total protein was quantified using the method described by Lowry et al. (1951). Sample volumes equivalent to 40 μg of protein were loaded onto a 12% SDS‐PAGE gel. After electrophoresis, proteins were transferred to nitrocellulose membranes. Staining with Ponceau S confirmed successful protein transfer. Membranes were washed with Tris‐buffered saline (TBS) and blocked with 5% skim milk in TBS for 1 h, followed by overnight incubation at 4°C with primary antibodies for CB1 receptor (1:1000, rabbit, #PA007048, Cusabio), CB2 receptor (1:1000, mouse, #WH0001269M, Sigma‐Aldrich), TRPV1 (1:1000, rabbit, #PA1‐29421, Invitrogen) and α‐tubulin (1:5000, mouse, #18023, Abcam). The following day, after TBS washes, membranes were incubated for 2 h at room temperature with horseradish peroxidase (HRP)‐conjugated secondary antibodies: HRP anti‐rabbit IgG (1:5000, goat, #A0545, Sigma‐Aldrich) or HRP anti‐mouse IgG (1:5000, goat, #A5278, Sigma‐Aldrich). Signals were developed using Immobilon Forte Western HRP substrate (Merck‐Millipore, WBLUF0500) and detected with a Chemidoc MP Imaging System (Bio‐Rad). All membranes were reused for analysis of multiple proteins; therefore, they were stripped with 0.2 M glycine buffer (pH 2.2) at 50°C for 20 min before a new round of blocking and antibody incubation. The incubation order was CB1 receptor, followed by CB2 receptor and TRPV1, and finally α‐tubulin. Protein bands were quantified using Image Lab software (Bio‐rad), and each protein's optical density was normalised to α‐tubulin levels. Full‐length blots for CB1 receptor, CB2 receptor, TRPV1 and α‐tubulin are provided in Figure S1.

### 2.9 Immunohistochemistry and Image Analysis

Fixed spinal cords were immersed sequentially in sucrose solutions of increasing concentrations (10%, 20% and 30%), for at least 24 h in each solution, and then sectioned in the cryostat into 14 μm‐thick coronal slices. Sections were washed 3 times with phosphate‐buffered saline (PBS) containing 0.5% Triton X‐100 (5 min each) and incubated with 5% normal goat serum and 5% horse serum for 1 h at room temperature. Primary antibody anti‐Iba‐1 (1:500, #234‐013, Synaptic Systems) was incubated overnight at 4°C. On the following day, after PBS washes, samples were incubated for 2 h at room temperature with Alexa 594‐conjugated goat anti‐rabbit IgG secondary antibody (1:500, # A21207, Life Technologies), followed by 4′,6‐diamidino‐2‐phenylindole (DAPI) staining (1:1000, #32670, Sigma‐Aldrich). Samples were mounted with coverslips using 40% glycerol in PBS (pH 7.4). Images from lamina I‐III from dorsal spinal cord horn were acquired using an ApoTome microscope (Zeiss Axio Imager M.2) with 20*×* objectives for microglial quantification and 40*×* objectives for microglial morphological analysis. For each animal, two to four slices were analysed, and mean values were used for statistical analysis. The number of microglia per area and the percentage of Iba‐1‐stained area were quantified using Zeiss Zen Blue 2.3 lite and FIJI 2.9 software, respectively. For microglial morphology, Sholl analysis was performed on 5–6 cells from 2 spinal cord slices per animal using FIJI 2.9 software.

### 2.10 Statistical Analysis

Statistical analyses were performed using GraphPad Prism 9.0. The normality of the data distribution was assessed with the Kolmogorov–Smirnov test, and potential outliers were identified by ROUT analysis. Outliers were excluded from further analysis. Behavioural test results were analysed using repeated‐measures two‐way ANOVA, followed by Tukey's post hoc test. Results from flow cytometry, western blotting and image analysis were analysed using one‐way ANOVA for normally distributed data, or the Kruskal‐Wallis test for non‐normally distributed data. Pre‐specified comparisons between groups sharing the same treatment or surgical condition were conducted using Šidák's or Dunn's post hoc tests. Data are expressed as mean ± SEM (standard error of the mean) or mean ± SD (standard deviation), as specified in the figure legends, and statistical significance was set at *p* < 0.05.

## 3 Results

### 3.1 Treatment With CBD‐Enriched *Cannabis* Extract Modestly Improved Cold Hyperalgesia in Rats of Both Sexes

Mechanical and thermal sensitivity thresholds were assessed in male and female rats at baseline (prior to sham surgery or CCI induction), 7 days post‐surgery (before treatment) and 21 days post‐surgery (after 14 days of daily administration of either the CBD‐enriched extract or vehicle).

**FIGURE 3:**
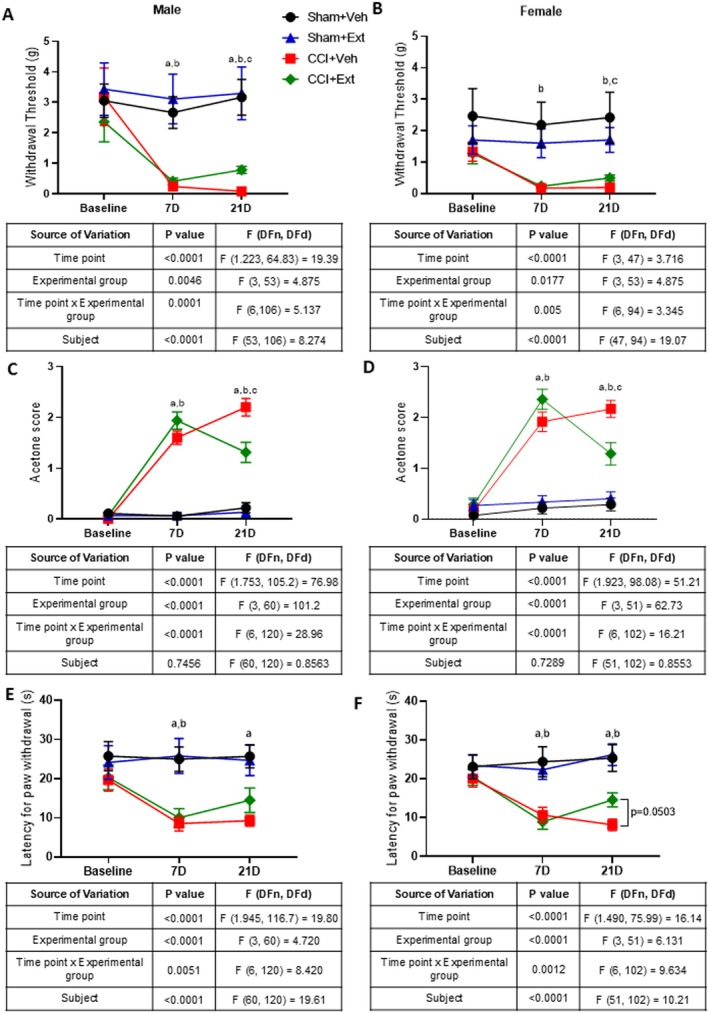
Treatment with CBD‐enriched Cannabis sp. extract moderately improves cold hyperalgesia, with a minor effect on mechanical allodynia and no effect on heat hyperalgesia, in male and female CCI rats. Mechanical thresholds evaluated by the von Frey test (A‐, B), cold sensitivity evaluated by the acetone test (C, D), and heat sensitivity evaluated by the hot plate test (E, F) in Sham and CCI male and female rats at baseline (pre‐surgery), 7 days after surgery (D), and 21 days after surgery (14 days after the start of daily oral administration of extract (Ext) or vehicle (Veh)). Statistical analysis was performed using repeated‐measures two‐way ANOVA followed by Tukey's post hoc test. Tables under each graph include *p* values, *F* values and degrees of freedom for each ANOVA. Letters in the graphs indicate post hoc comparisons: a = Sham + Veh versus CCI + Veh *p* < 0.05, b = Sham + Ext versus CCI + Ext *p* < 0.05, c = CCI + Veh versus CCI + Ext *p* < 0.05. Data are presented as mean ± standard error of the mean; *n* = 12–16 animals per experimental group.

Male and female rats subjected to CCI exhibited reduced mechanical thresholds compared with their respective SHAM groups 7 days after surgery, indicating the development of mechanical allodynia (males: Sham + Veh vs. CCI + Veh, *p* = 0.0024; Sham + Ext vs. CCI + Ext, *p* = 0.0282; females: Sham + Veh vs. CCI + Veh, *p* = 0.0729; Sham + Ext vs. CCI + Ext, *p* = 0.0395). By Day 21, the 2‐week treatment with the CBD‐enriched extract led to a statistically significant increase in mechanical thresholds in CCI rats compared with vehicle‐treated CCI animals in both males (*p* = 0.0006) and females (*p* = 0.0367). However, the treatment did not fully reverse mechanical allodynia, as thresholds in CCI animals remained lower than those of their Sham groups (males: SHAM + Veh vs. CCI + Veh, *p* = 0.0006; SHAM + Ext vs. CCI + Ext, *p* = 0.0576; females: SHAM + Veh vs. CCI + Veh, *p* = 0.0755; SHAM + Ext vs. CCI + Ext, *p* = 0.0428) (Figure 3A,B). Furthermore, inspection of the mean mechanical thresholds in CCI + Veh and CCI + Ext groups in males (0.086 g and 0.79 g, respectively) and females (0.192 g and 0.498 g, respectively) suggests a limited functional impact of the treatment.

We next evaluated cold sensibility using the acetone test. Both male and female CCI animals showed increased scores compared with their respective Sham groups 7 days after surgery, indicating the development of cold hyperalgesia (males: SHAM + Veh vs. CCI + Veh, *p* < 0.0001; SHAM + Ext vs. CCI + Ext, *p* < 0.0001; females: SHAM + Veh vs. CCI + Veh, *p* < 0.0001; SHAM + Ext vs. CCI + Ext, *p* < 0.0001). CBD‐enriched extract significantly reduced acetone test scores in CCI animals compared with vehicle‐treated CCI rats 14 days after treatment, in both males (*p* = 0.0112) and females (*p* = 0.0199). However, the treatment did not reverse cold hyperalgesia completely, since CCI + Ext animals still exhibited significantly higher scores than their respective Sham groups in both sexes (males: SHAM + Veh vs. CCI + Veh, *p* < 0.0001; SHAM + Ext vs. CCI + Ext, *p* = 0.0001; females: SHAM + Veh vs. CCI + Veh, *p* < 0.0001; SHAM + Ext vs. CCI + Ext, *p* = 0.0116) (Figure 3C,D).

The hot plate test was then used to evaluate thermal pain thresholds at high temperatures. Rats subjected to CCI had shorter paw withdrawal latencies compared to Sham animals 7 days after surgery, indicating the development of thermal hyperalgesia (males: SHAM + Veh vs. CCI + Veh, *p* = 0.0006; SHAM + Ext vs. CC I+ Ext, *p* = 0.0247; females: SHAM + Veh vs. CCI + Veh, *p* = 0.0236; SHAM + Ext vs. CCI + Ext, *p* = 0.0012). Unlike the effects observed on mechanical and cold sensitivity, treatment with the CBD‐enriched extract did not significantly improve heat thresholds in the hot plate test in CCI animals compared with the CCI + Veh group at Day 21, although females showed a trend toward significance (males: *p* = 0.4454; females: *p* = 0.0503). Male CCI + Veh animals continued to exhibit significantly shorter paw withdrawal latencies relative to SHAM + Veh rats (males: SHAM + Veh vs. CCI + Veh, *p* = 0.002; SHAM + Ext vs. CCI + Ext, *p* = 0.2). In females, both vehicle‐ and extract‐treated CCI groups had significantly shorter latencies than theirs respective Sham controls (females: SHAM + Veh vs. CCI + Veh, *p* = 0.0014; SHAM + Ext vs. CCI + Ext, *p* = 0.0092) (Figure 3E,F).

These results demonstrate that daily treatment with a CBD‐enriched *Cannabis* extract moderately reduced cold hyperalgesia in rats of both sexes, with no significant modulation of high‐temperature thresholds. Regarding mechanical allodynia, although statistically significant, the magnitude of the change was small and likely of limited relevance in terms of pain relief.

### 3.2 The Increase in Spinal Cord Microglial Cell Number in Female CCI Rats Is Not Modulated by the CBD‐Enriched Extract

**FIGURE 4:**
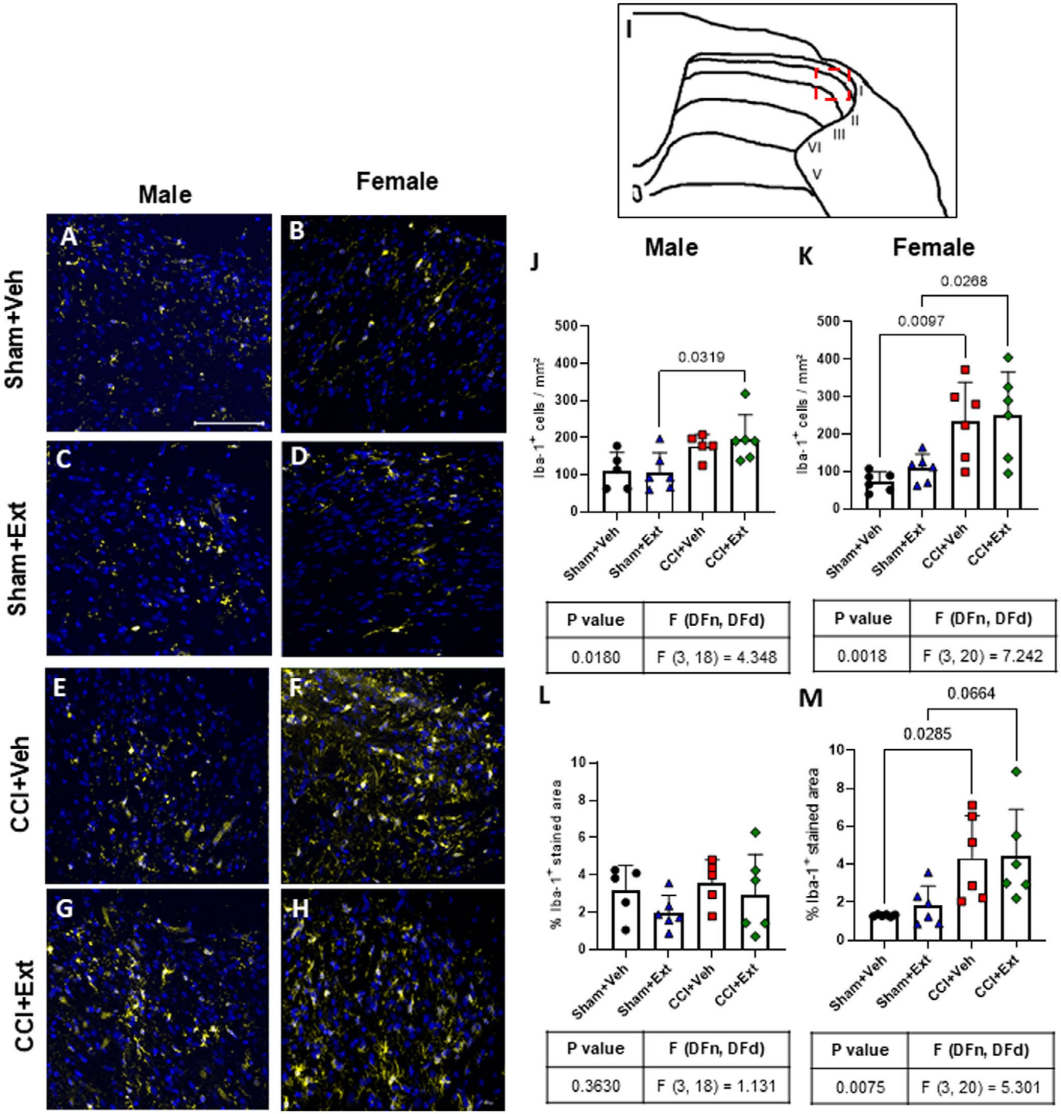
CCI increases microglial cell numbers in the dorsal spinal cord of female rats, without modulation by CBD‐enriched extract. Representative photomicrographs of males (A, C, E, G) and females (B, D, F, H), and an image of laminae I‐III of the ipsilateral spinal cord indicating the analysed region (red square) (I). Quantification of the number of Iba‐1 positive cells (J, K) and the percentage of the Iba‐1‐stained area (L, M) in male and female rats 22 days after surgery. Data were analysed using one‐way ANOVA followed by Šidák's post hoc test. Tables under each graph summarise *p* values, *F* values and degrees of freedom from ANOVA tests. Values are expressed as mean ± standard deviation; *n* = 5–6 animals per group. Scale bar = 100 μm.

Microglial cells are considered a key element in the development of CNP (IInoue and Tsuda 2018; Tsuda 2019). The number of Iba‐1‐positive cells and the percentage of Iba1‐stained area were quantified in the dorsal horn of the spinal cord (lamina I‐III) 22 days post‐surgery (Figure 4I—red square). In males, a significant increase in Iba‐1‐positive cells was observed only in the CCI + Ext group compared with its respective Sham group (Sham + Ext vs. CCI + Ext *p* = 0.0319), whereas no difference was observed between Sham and CCI animals in the vehicle‐treated group (Sham + Veh vs. CCI + Veh *p* = 0.788) (Figure 4J). In females, CCI rats from both vehicle‐ and extract‐treated groups showed significantly higher numbers of Iba‐1 positive cells (Sham + Veh vs. CCI + Veh *p* = 0.0097 and Sham + Ext vs. CCI + Ext *p* = 0.0268) and greater Iba1‐stained area compared with their respective Sham controls (Sham + Veh vs. CCI + Veh *p* = 0.0285 and Sham + Ext vs. CCI + Ext *p* = 0.0664) (Figure 4K,M). Male rats showed no difference in Iba1‐stained area between Sham and CCI groups (Sham + Ext vs. CCI + Ext *p* = 0.727, Sham + Veh vs. CCI + Veh *p* = 0.986).

No differences were detected when treated CCI or Sham animals were compared with their untreated controls in either sex (Figure 4J–M).

**FIGURE 5:**
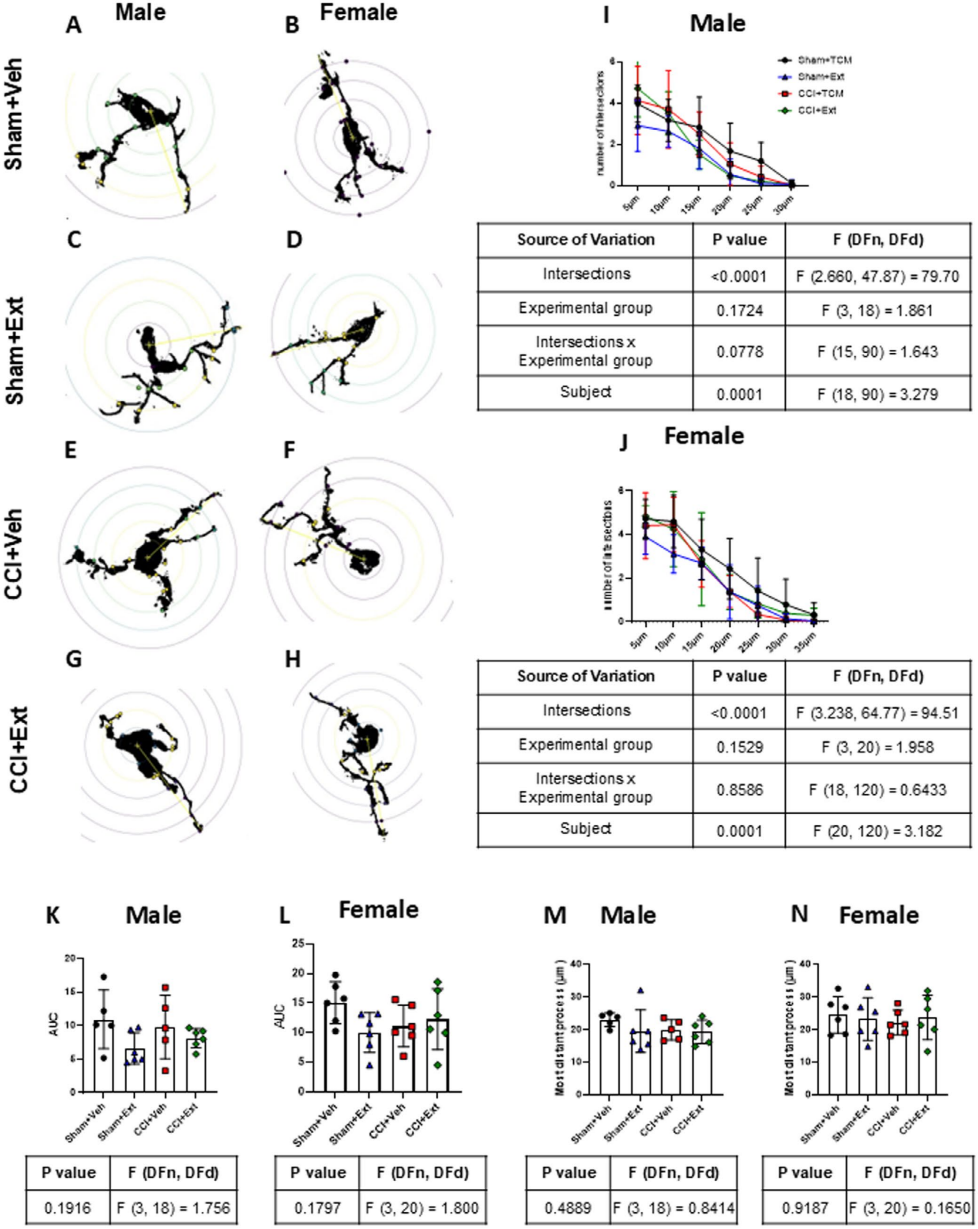
Microglial Sholl analysis reveals no morphological changes in dorsal spinal cord Iba‐1‐positive cells across experimental groups 22 days after surgery. Representative images of analysed microglial cells (A–H). Quantification of the number of intersections at 5 μm intervals from the cell body (I–J), the area under the curve derived from the intersection graphs (K, L), and the maximum projection length (M, N). Data were analysed using two‐way ANOVA followed by Tukey's post hoc test (I and J) or one‐way ANOVA followed by Šidák's post hoc test (K–N). Tables under the graphs show *p* values, *F* values and degrees of freedom from ANOVA tests. Values are expressed as mean ± standard deviation; *n* = 5–6 animals per experimental group.

Alterations in microglial morphology may reflect changes in their phenotype, as a consequence of cellular responses to different types of pathological stimuli (Midavaine et al. 2021). Sholl analysis was performed on Iba‐1‐positive cells in laminae I‐III of the ipsilateral spinal cord from male and female rats across all experimental groups (Figure 5). No significant differences were observed in the number of intersections (Figure 5I,J), the area under the curve of the intersection graph (Figure 5K,L), or the distance of the most distant projection (Figure 5M,N) when comparing CCI groups with their respective Sham groups in either sex. Similarly, no differences were found when CCI or Sham animals treated with the CBD‐enriched Cannabis sp. extract were compared with their untreated controls, regardless of sex (Figure 5I–N).

These results indicate that CCI increased microglial cell number in the spinal cord of female rats, and that the 2‐week treatment with the CBD‐enriched *Cannabis* sp. extract had no detectable effect on this response. No consistent changes were observed in microglial morphology at this time point, regardless of treatment.

### 3.3 Flow Cytometry Analysis of the Ipsilateral Spinal Cord 22 Days After Surgery Shows no Infiltration of Peripheral Immune Cells

One of the main pathophysiological features of CNP is the infiltration of circulating immune cells, such as T lymphocytes, into the spinal cord (Hu et al. 2007; Sorge et al. 2015; Du et al. 2018). We therefore performed flow cytometry analysis of immune cells in the ipsilateral spinal cord 22 days after CCI or Sham surgery. Our results revealed that microglial cells are the main contributors to the immune responses at this time point, with no apparent major contribution from infiltrating lymphocytes or neutrophils.

**FIGURE 6:**
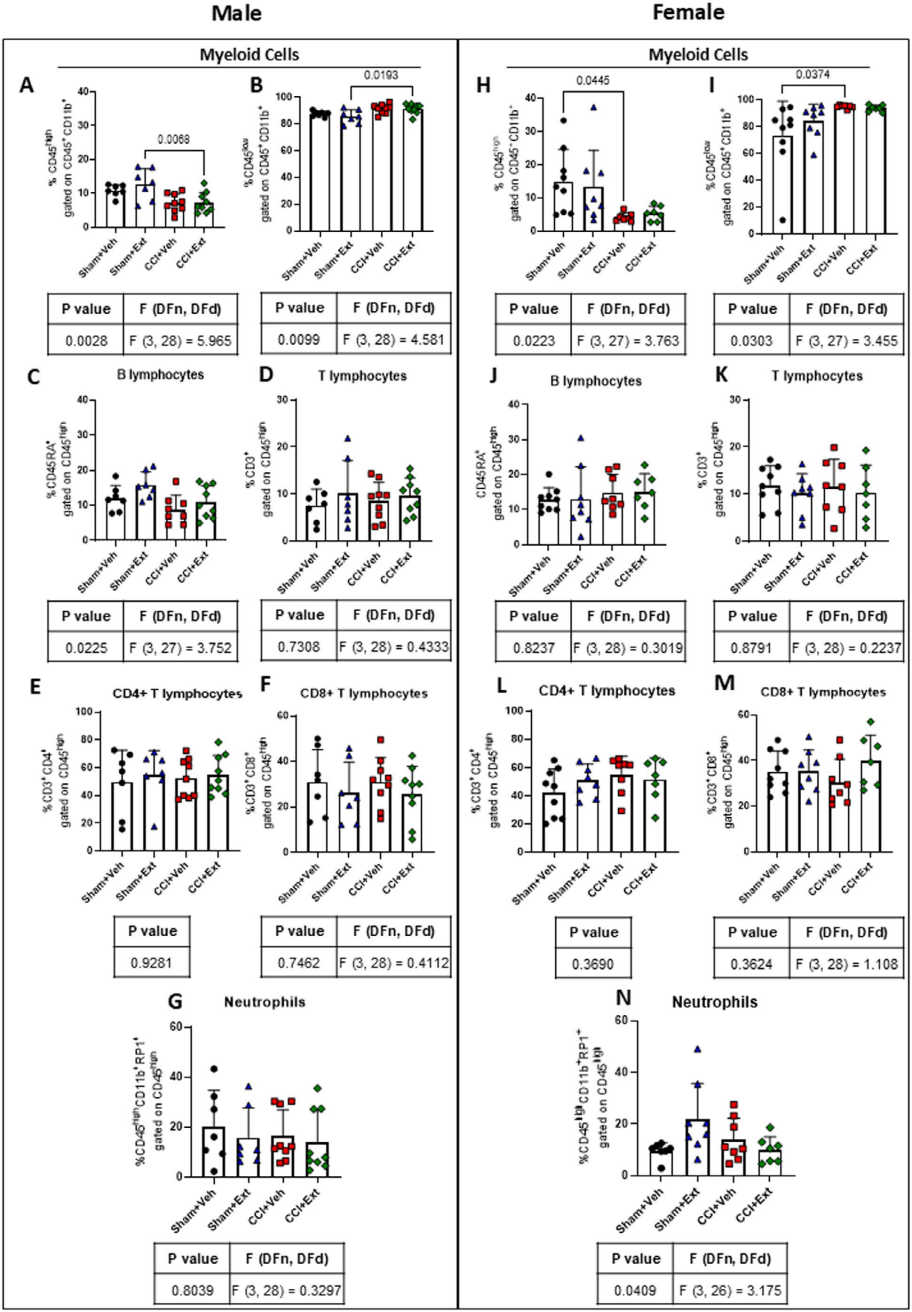
CCI induces changes in the percentage of the CD45^low^CD11b^+^ microglial cell population in the spinal cord, without peripheral immune cell infiltration, at 22 days post‐surgery. Flow cytometry analysis of the percentages of innate and adaptive immune cells in the ipsilateral spinal cord of male (A–G) and female (H–N) rats 22 days post‐surgery. Data were analysed using one‐way ANOVA or the Kruskal‐Wallis test, followed by Šidák's or Dunn's post hoc tests. Tables under the graphs present *p* values, *F* values and degrees of freedom from ANOVA tests, as well as *p* values, *H* values and degrees of freedom from the Kruskal‐Wallis tests. Values are expressed as mean ± standard deviation; *n* = 7–9 animals per experimental group.

Male CCI rats showed a decrease in the CD45^high^CD11b^+^ population, mainly composed of macrophages and infiltrating myeloid cells (Sham + Ext vs. CCI + Ext *p* = 0.0068), and an increase in CD45^low^CD11b^+^ microglia (Sham + Ext vs. CCI + Ext *p* = 0.0193) when comparing Sham and CCI animals treated with the CBD‐enriched *Cannabis* extract (Figure 6A,B). Female rats showed a similar pattern, with an increase in the microglial population that reached statistical significance when comparing Sham and CCI animals treated with vehicle (CD45^high^ Sham + Veh vs. CCI + Veh *p* = 0.0445 and CD45^low^ Sham + Veh vs. CCI + Veh *p* = 0.0374) (Figure 6H,I). Importantly, no significant differences were observed between CBD‐enriched extract‐treated and vehicle‐treated groups (Figure 6).

Additionally, no differences were observed in the infiltration of B lymphocytes (Figure 6C,J), T lymphocytes (Figure 6D,K) and their CD4^+^ and CD8^+^ subsets (Figure 6E,F,L,M), or neutrophils (Figure 6G,N), either between CCI and Sham groups or between CBD‐enriched extract‐treated and vehicle‐treated groups.

These data indicate that CCI induces changes in the CD45^low^CD11b^+^ microglial population 22 days after surgery, when the pain was already established. However, we did not find evidence of peripheral immune cell infiltration at this time point, regardless of treatment.

### 3.4 CCI Does Not Modulate the Expression of Endocannabinoid Receptors in the Ipsilateral Spinal Cord, Independently of Treatment Condition

**FIGURE 7:**
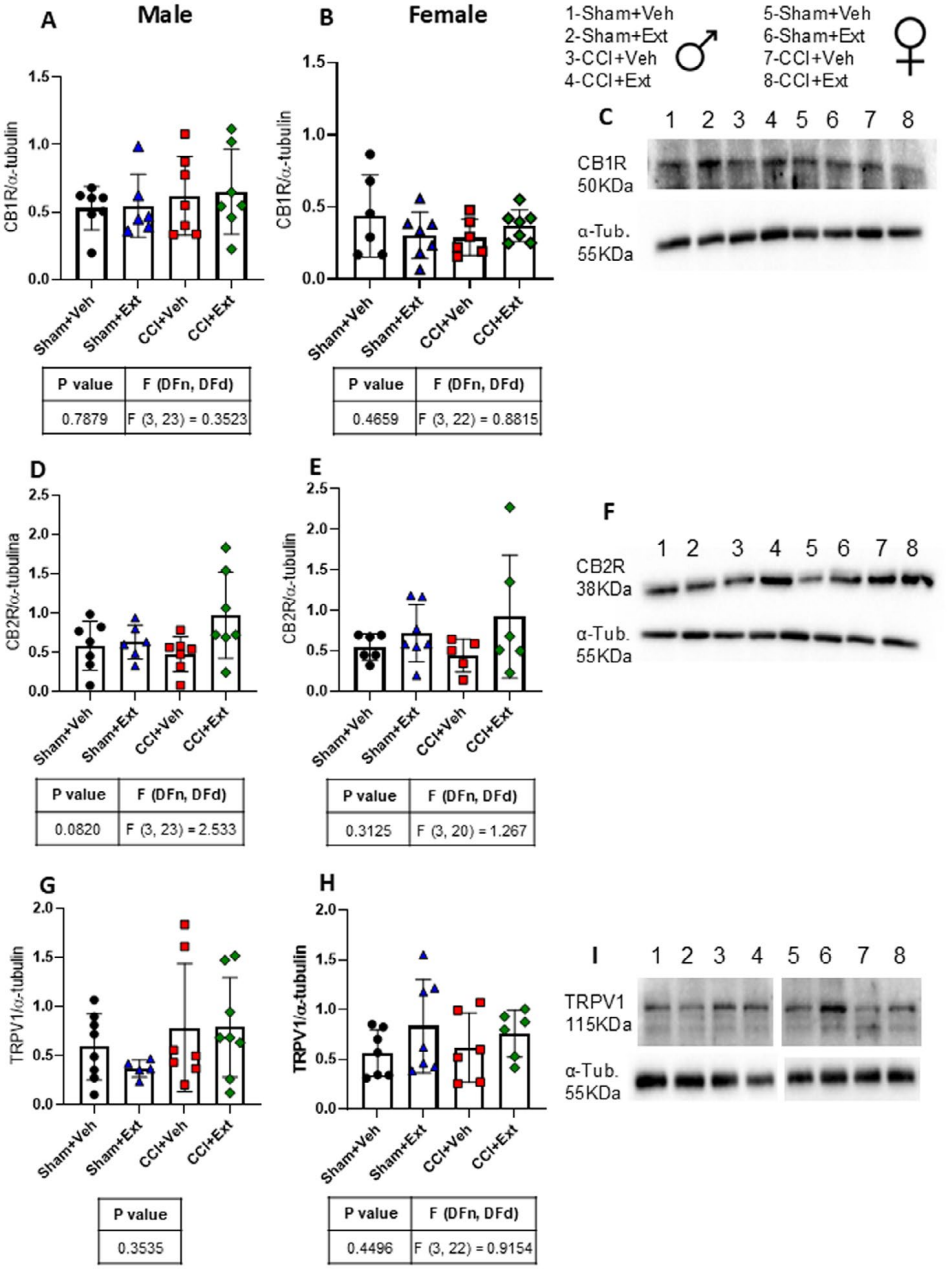
CB1R, CB2R and TRPV1 levels are not altered in the ipsilateral spinal cord 22 days after CCI or Sham surgery, regardless of treatment. Quantification histograms and representative immunoblot images showing the expression levels of CB1R (A–C), CB2R (D–F) and TRPV1 (G–I), normalised to α‐tubulin, in the ipsilateral spinal cord of male and female rats 22 days after surgery. Data were analyzed using one‐way ANOVA or the Kruskal–Wallis test, followed by Šidák's or Dunn's post hoc tests. Tables under the graphs present *p* values, *F* values and degrees of freedom from ANOVA tests, as well as the *p* value, *H* value and degree of freedom from the Kruskal‐Wallis test. Values are expressed as means ± standard deviation; *n* = 6–7 animals per experimental group.

The EcS plays a key role in the regulation of nociception and is one of the major signalling systems modulated by phytocannabinoids and terpenes. Protein levels of CB1R, CB2R and TRPV1 were assessed in the ipsilateral spinal cord 22 days post‐surgery (Figure 7). There were no statistically significant differences in CB1R, CB2R, or TRPV1 expression between experimental groups in either male (Figure 7A,D,G) or female rats (Figure 7B,E,H).

These findings indicate that neither chronic neuropathic pain nor treatment with the CBD‐enriched extract alters the expression of the main endocannabinoid receptors involved in nociception regulation 22 days after the induction of CNP.

## 4 Discussion

There is a growing medical interest in the use of *Cannabis* sp.‐based products, particularly *Cannabis* sp. extracts, for the treatment of several neurological diseases, including epilepsy and autism spectrum disorders (Gaston and Szaflarski 2018; Paes‐Colli et al. 2022). However, the IASP published findings from a task force study on Cannabis and cannabinoids, concluding that the society cannot endorse the use of *Cannabis* sp.‐based products for chronic pain treatment. This conclusion was based on the lack of rigorous scientific data and significant gaps in the translational spectrum, particularly concerning the mechanisms of action underlying cannabinoid‐mediated analgesia (Haroutounian et al. 2021).

CNP shows sex differences in incidence, and mechanisms underlying this condition differ between males and females. These differences include the participation of immune cells in the CNS and modulation of the EcS (Ruau et al. 2012; Mogil 2020; Midavaine et al. 2021; Blanton et al. 2021; Keogh 2022). Nevertheless, many preclinical studies, including those in the field of cannabinoids and pain research, rely exclusively on male subjects in their experimental designs (Comelli et al. 2009; Pearl‐Dowler et al. 2023; Islas‐Espinoza et al. 2025). Another important consideration is that preclinical studies investigating the effects of *Cannabis* sp.‐based products often fail to replicate the routes of administration commonly used by patients (King et al. 2017; Casey et al. 2017; Rouhollahi et al. 2020) and rarely explore the cellular or molecular mechanisms behind the behavioral outcomes. Notably, our study addressed some of these gaps: it included animals of both sexes and employed a clinically relevant oromucosal route of administration.

Our results demonstrate that daily oromucosal treatment with a CBD‐enriched extract for 2 weeks (10 mg/kg/day), initiated 7 days after CCI surgery, when CNP symptoms were already established, can partially improve cold thermal hyperalgesia in rats of both sexes, with a minor effect on mechanical allodynia. However, these responses did not return to baseline values or reach those observed in Sham animals, in agreement with other preclinical studies (Comelli et al. 2009; Mabou Tagne et al. 2021). Clinical reports also indicate that treatment with *Cannabis* sp. products, especially full extracts containing several phytocannabinoids and other components, often yields partial pain relief, although clinical data still require more rigorous studies (Ueberall et al. 2019; O'Brien et al. 2023; Chou et al. 2025).

No significant improvement was observed in the hot plate test following treatment, although results in female rats approached statistical significance. Comelli et al. (2009) reported an improvement in high‐temperature thermal hyperalgesia in rats with diabetic neuropathy after 8 days of daily treatment with a CBD‐enriched *Cannabis* sp. extract at approximately 30 mg/kg CBD. Notably, no such improvement was observed at a dose of 15 mg/kg CBD. These findings suggest that modulation of heat sensitivity may be less responsive to low‐dose cannabinoid exposure. In this context, the absence of a significant effect in the hot plate test, together with the modest magnitude of the effects on mechanical allodynia, may reflect the conservative dosing strategy employed. The 10 mg/kg CBD dose was intentionally selected to evaluate a low, clinically relevant exposure while minimising THC intake from the full‐spectrum extract, thereby reducing the risk of psychoactive effects. Importantly, this dose falls within the range previously shown to produce behavioural and cellular effects in models of chronic pain and neurological disorders (Comelli et al. 2008; Silva‐Cardoso et al. 2021), whereas lower doses (2.5–5 mg/kg) have been reported to be largely ineffective in modulating nociception and cognitive outcomes (Costa et al. 2007; Fagherazzi et al. 2012). Moreover, considering the pharmacokinetic profile of CBD, the dosing regimen used in the present study is likely to have produced biologically relevant exposure, despite once‐daily administration. Previous work has shown that oral CBD at 10 mg/kg in Sprague–Dawley rats reaches peak plasma concentrations within 2 h, followed by a gradual decline over a 24‐h period (Schwotzer et al. 2023). In addition, CBD accumulates in nervous tissue following oral administration (20 mg/kg), with concentrations persisting in the brain even after plasma levels decline, supporting prolonged pharmacological activity at target sites (Dumbraveanu et al. 2023).

Here, we investigated whether treatment with a CBD‐enriched extract could modulate neuroinflammation following CCI. Several studies have reported that peripheral immune cells are involved in the development of CNP, such as T lymphocytes that infiltrate the spinal cord 3–10 days after CNP induction (Hu et al. 2007; Cao and DeLeo 2008; Costigan et al. 2009; Lewis 2013; Li et al. 2020; Sorge et al. 2015; Tsuda 2019). However, flow cytometry analysis showed no evidence of immune cell infiltration in the spinal cord related to the CCI model or to treatment. Most available data on immune cell infiltration in the spinal cord in this condition refer to acute time points. Thus, the lack of alterations in these cell populations may be related to the relatively late time point evaluated (22 days post‐surgery).

Microglial cells in the spinal cord are widely recognised as key mediators of mechanical allodynia in neuropathic pain models, especially through the release of cytokines and trophic factors, such as brain‐derived neurotrophic factor (IInoue and Tsuda 2018; Tsuda 2019). Flow cytometry and immunofluorescence analyses revealed that CCI increased the presence of microglial cells in the spinal cord 22 days after surgery compared to Sham animals, particularly in females. In males, however, this difference was not statistically significant between the Sham + Veh and CCI + Veh groups. This lack of statistical significance could be due to the limited power to detect small differences at this time point, since previous studies have reported a decrease in microgliosis in the spinal cord from 1–2 to 3–4 weeks after induction of chronic pain (Zhang and De Koninck 2006; Shu et al. 2020; Mussetto et al. 2023). These time‐dependent changes in microglial reactivity may also explain the absence of detectable alterations in microglial morphology between Sham and CCI groups. Mussetto et al. (2023) observed changes in microglial morphology when comparing Sham to CCI male rats 1 week after surgery; however, these alterations were not observed at 4 and 7 weeks post‐CNP induction. Finally, although we hypothesised that the CBD‐enriched extract could modulate neuroinflammatory pathways due to the known anti‐inflammatory properties of phytocannabinoids, we did not detect treatment‐related changes in microglial number in females.

Several molecular targets have been identified through which the CBD‐enriched *Cannabis* sp. extract may influence behavioural outcomes, including CB1R, CB2R and TRPV1. THC acts as a partial agonist of both CB1R and CB2R, whereas CBD acts as an agonist of TRPV1 (Patricio et al. 2020). Rapid desensitisation of the TRPV1 channel leads to analgesic and anti‐inflammatory effects. This action underlies some of CBD's pain‐relieving properties, making TRPV1 a key target for therapeutic research in pain and inflammation (Bisogno et al. 2001).

An indirect way by which the extract could modulate these receptors is through changes in endocannabinoid levels, since CBD inhibits FAAH, the enzyme responsible for degrading AEA, an endogenous agonist of CB1R and CB2R (Watanabe et al. 1996; Bisogno et al. 2001; De Petrocellis et al. 2011). However, we did not find any significant modulation of CB1R, CB2R, or TRPV1 expression, either as a result of CCI or following treatment with the CBD‐enriched extract. Although previous studies have described upregulation of CB2R in the spinal cord in CNP models, these findings were observed at earlier time points (7–15 days after CNP induction), whereas our analyses were conducted 22 days after CCI (Zhang et al. 2003; Walczak et al. 2005; Romero‐Sandoval and Eisenach 2007; Racz et al. 2008). The absence of significant modulation of CB1R and CB2R expression by the treatment is consistent with previous findings in the hippocampus of healthy rats treated daily for 2 weeks with 3 mg/kg of a CBD‐enriched *Cannabis* sp. extract (Aguiar et al. 2023).

Taken together, our data suggest that 2‐week administration of a CBD‐enriched *Cannabis* sp. extract partially alleviates cold hypersensitivity in a rat model of neuropathic pain. At the neuroimmune level, CCI induced alterations predominantly in spinal cord microglial cells, whereas no detectable changes were observed in infiltrating lymphocytes or neutrophils at the late time point studied, and no treatment‐related effects were identified. In addition, neither treatment with the CBD‐enriched Cannabis sp. extract nor CCI surgery altered endocannabinoid receptor expression in the spinal cord at the time point evaluated. A limitation of this study is that only a single dose of the CBD‐enriched extract was tested and that neuroimmune alterations were evaluated at a single time point. Future studies should evaluate earlier post‐injury time points to capture acute and transient neuroimmune changes, as well as investigate dose–response relationships.

## 5 Conclusion

Administration of a CBD‐enriched *Cannabis* sp. extract daily for 2 weeks has a modest effect on neuropathic pain symptoms in rats of both sexes, partially attenuating cold hypersensitivity. This effect occurs without detectable modulation of microgliosis in females. Moreover, at the time point studied, we did not detect immune cell infiltration or changes in the expression of endocannabinoid receptors in the spinal cord, regardless of treatment condition.

## Author Contributions

**Raquel Maria Pereira Campos:** project conceptualisation, methodology, experiments, data analysis and manuscript preparation. **Luciana Conde:** methodology, experiments and flow cytometry analysis. **Andrey Aguiar:** methodology, experiments, phytocannabinoids analysis and animal handling and treatment. **Luzia Sampaio:** project conceptualisation, reviewing and editing. **Ricardo Augusto de Melo Reis:** project conceptualisation, supervision, reviewing and editing. **Pedro Moreno Pimentel‐Coelho:** project conceptualisation, data analysis and interpretation, supervision, reviewing and editing.

## Funding

This work was supported by the Coordenação de Aperfeiçoamento de Pessoal de Nível Superior (CAPES) (Grant 88887.360870/2019‐00), the Fundação de Amparo a Pesquisa do Estado do Rio de Janeiro (FAPERJ) (Grant E‐26/201.279/2021, E‐26/200.014/2024, 200.015/2024 and E‐26/204.252/2024) and the Conselho Nacional de Desenvolvimento Científico e Tecnológico (CNPq) (Grants 313757/2020‐8 and 311188/2023‐0).

## Disclosure

The CBD‐enriched *Cannabis* sp. extract and MCT were obtained from HempMeds under a simplified special authorisation for research institutions granted by the Brazilian National Health Surveillance Agency (ANVISA) (Authorization No. 013/2021).

## Conflicts of Interest

The authors declare no conflicts of interest.

## Supporting information


**Figure S1:** Full‐length blots of CB1R (A), CB2R (B), TRPV1 (C) and respective and α‐tubulin staining used for quantification.
