# Supplementary material for: Oromucosal Administration of a Cannabidiol‐Enriched Cannabis sp. Extract for 2 Weeks Moderately Reduces Cold Hyperalgesia in Rats With Neuropathic Pain
Source: Eur J Pain. 2026 Jun 5;30(6):e70307. doi: 10.1002/ejp.70307 (PMC13241296; doi:10.1002/ejp.70307)
Supplement: Supplementary file 1 — Figure S1: Full‐length blots of CB1R (A), CB2R (B), TRPV1 (C) and respective and α‐tubulin staining used for quantification. [file EJP-30-0-s001.docx]

Supplementary Figure 1


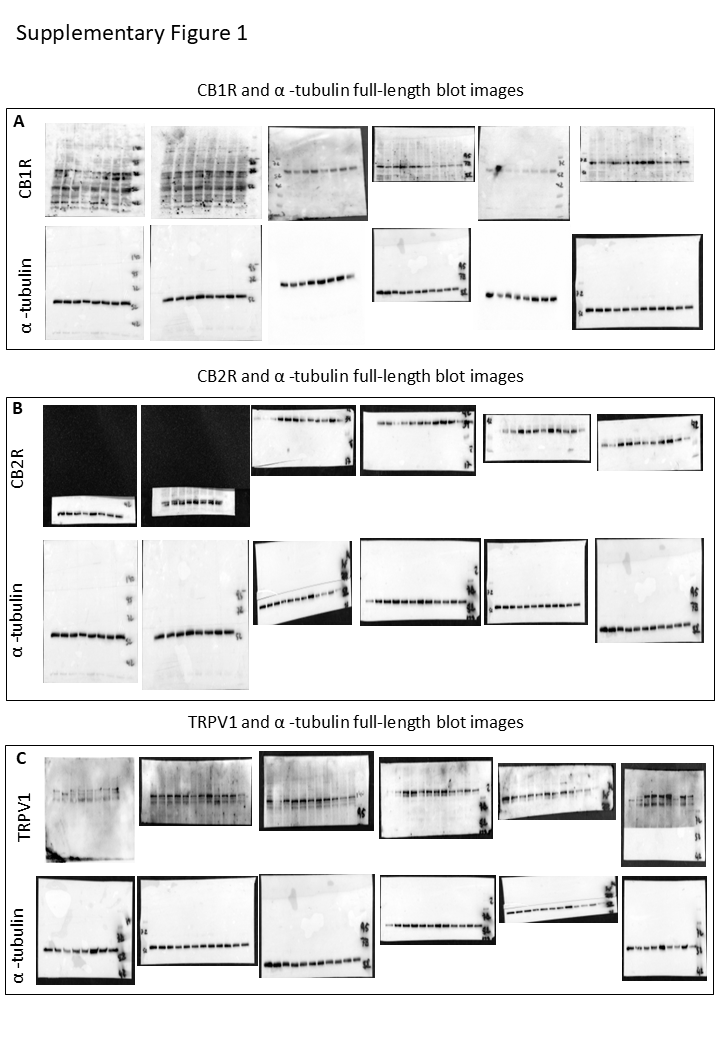


Figure S1: Full-length blots of CB1R (A), CB2R (B), TRPV1 (C) and respective and α-tubulin staining used for quantification.
